# Occupational Exposure of Diesel Station Workers to BTEX Compounds at a Bus Depot

**DOI:** 10.3390/ijerph120404101

**Published:** 2015-04-13

**Authors:** Raeesa Moolla, Christopher J. Curtis, Jasper Knight

**Affiliations:** School of Geography, Archaeology and Environmental Studies, University of the Witwatersrand, Private Bag X3 WITS, Gauteng 2050, South Africa; E-Mails: Christopher.Curtis@wits.ac.za (C.J.C.); Jasper.Knight@wits.ac.za (J.K.)

**Keywords:** diesel, BTEX, health risk assessment, lifetime cancer risk, hazard quotient

## Abstract

Diesel fuel is known to emit pollutants that have a negative impact on environmental and human health. In developing countries like South Africa, attendants are employed to pump fuel for customers at service stations. Attendants refuel vehicles with various octane unleaded fuel, lead-replacement petrol and diesel fuel, on a daily basis. Attendants are at risk to adverse health effects associated with the inhalation of volatile organic compounds released from these fuels. The pollutants released include benzene, toluene, ethylbenzene and xylenes (BTEX), which are significant due to their high level of toxicity. In this study, a risk assessment of BTEX was conducted at a diesel service station for public buses. Using Radiello passive samplers, it was found that benzene concentrations were above recommended international standards. Due to poor ventilation and high exposure duration, the average benzene concentration over the sampling campaign exceeded the US Environmental Protection Agency’s chronic inhalation exposure reference concentration. Lifetime cancer risk estimation showed that on average there is a 3.78 × 10^−4^ cancer risk, corresponding to an average chronic daily intake of 1.38 × 10^−3^ mg/kg/day of benzene exposure. Additionally, there were incidences where individuals were at potential hazard risk of benzene and toluene that may pose non-carcinogenic effects to employees.

## 1. Introduction

Inhalation of pollutants such as volatile organic compounds (VOCs) has been shown to have many side-effects on human health. A group referred to as BTEX (benzene, toluene, ethylbenzene and the three isomers of xylene) has been found to be potentially hazardous to environmental and human health [1]. Human exposure to BTEX, both through inhalation or ingestion, can have serious health impacts, such as neurological diseases, cancers, and teratogenic effects [2,3,4]. This is of major concern, as Chauhan *et al.* [2] state that 50% of BTEX inhaled by humans over a person’s lifespan is actually absorbed into the body. The World Health Organization (WHO) estimate that 4 in 1 million people are at risk of developing leukemia in their lifetime when exposed to 1 mg/m^3^ of benzene [5].

In South Africa, as is the case in many developing countries, people are still employed to refuel vehicles, trucks and buses at gas stations. In South Africa petrol pump attendants refuel vehicles with lead replacement petrol (LRP); 93-unleaded and 95-unleaded petrol; and 200, 50, or 10 ppm sulphur diesel, on a daily basis [6]. As such, attendants are particularly at risk to adverse health effects associated with inhalation of hazardous air pollutants (HAPs), such as BTEX which are released from these fuels. These attendants are thus exposed to both petrol and diesel fumes daily. However, despite numerous studies investigating the effects of gasoline inhalation on petrol pump workers and auto mechanics, there are no health risk assessment (HRA) studies focusing on diesel pump workers, despite most retail garages in developing countries providing both petrol and diesel services (Table 1). The purpose of this paper is to perform a site-specific health risk analysis to investigate the occupational exposure to BTEX, and inhalation risk, of workers at a diesel refueling station in South Africa. As many auto mechanics also face the risk of adverse health effects when exposed to BTEX, the health analysis will focus on both diesel pump attendants, as well as auto mechanics at a bus depot.

**Table 1:** A review of health risk assessments (HRAs), of various volatile organic compounds (VOC), specifically benzene, toluene, ethyl-benzene and xylenes (BTEX) studies, and conducted at/near petrol (gasoline) filling stations, in chronological date order. (BTX—Benzene, toluene and xylenes).

Location	Focus Area	Sampling Method	Ref.
Rangoon, Burma	Occupational benzene exposure in petrol filling stations	Urine samples	[7]
Kanpur/Lucknow, India	Environmental impact on health of workers at retail petrol pumps	Rotheroe and Mitchell personal samplers	[8]
Mexico City	Environmental exposure to VOCs among workers	Passive organic vapour badges and blood samples	[9]
Prunay, France	BTX concentrations near a stage II implemented petrol station	Gas chromatography + flame ionisation detector	[10]
--	Occupational exposure to benzene in gasoline filling station attendants	Radiello passive samplers and urine samples	[11]
Valencia, Spain	Air quality of BTEX inside vehicles and at gasoline filing stations	semipermeable membrane devices	[3]
Ioannina, Greece	Ambient benzene concentrations in the vicinity of petrol stations and associated health risk	Passive and active samplers	[12]
Ioannina, Greece	Assessment and prediction of exposure to benzene of filling station employees	Active and passive samplers	[13]
Chonburi, Thailand	HRA of VOCs in gas service station workers	Urine samples and air samplers	[14]
Kolkata, India	VOCs at petrol pumps: Exposure of workers and HRA	Personal air samplers	[15]
Calaba, Nigeria	Exposure of petrol station attendants and auto mechanics to petrol fumes	Structured questionnaires, venous blood samples analysis	[6]
Hyderabad, India	Geno-toxicity of filling station attendants exposed to petroleum hydrocarbons	Blood samples and Comet Assay	[16]
Murcia, Spain	Assessing the impact of petrol stations on their immediate surroundings	Radiello passive samplers	[17]
Montreal, Canada	BTEX exposures in automobile mechanics and health risks	Active chemical ionisation mass spectrometry	[18]
Bangkok, Thailand	Occupational exposure of gasoline station workers to BTEX compounds	Active samplers	[19]
India	Occupational health exposure at petroleum refinery	Organic vapour samplers	[20]
Bangkok, Thailand	HRA of petrol station workers and assessing exposure of inhaling BTEX	Personal air samplers	[21]
Multiple areas	HRA of BTX in gasoline service stations	BTX exposure data from scientific literature	[22]
Australia	Leukaemia and exposure to benzene in petroleum workers	Diagnostic information	[23]
Johannesburg, South Africa	Air quality of BTEX at a diesel filing station	Gas chromatography + photo ionisation detector	[24]

As a case study, this work is applied to a bus depot where there is significant movement of diesel buses. This analysis can thus provide useful information about potential health risks associated with BTEX vapours released from diesel refueling pumps and exhaust emissions.

## 2. Experimental Section

### 2.1. Study Site

The monitoring campaign was carried out at a bus depot located in central Johannesburg, South Africa. A government owned entity, which supplies the public transportation routes in central and northern Johannesburg, manages the depot. The bus depot accommodates 400 buses, where refueling, repairs and general maintenance of the buses are handled. The operating hours of the refueling bay and adjacent workshop is from 07:30 to 15:30, Monday–Friday.

**Figure 1:**
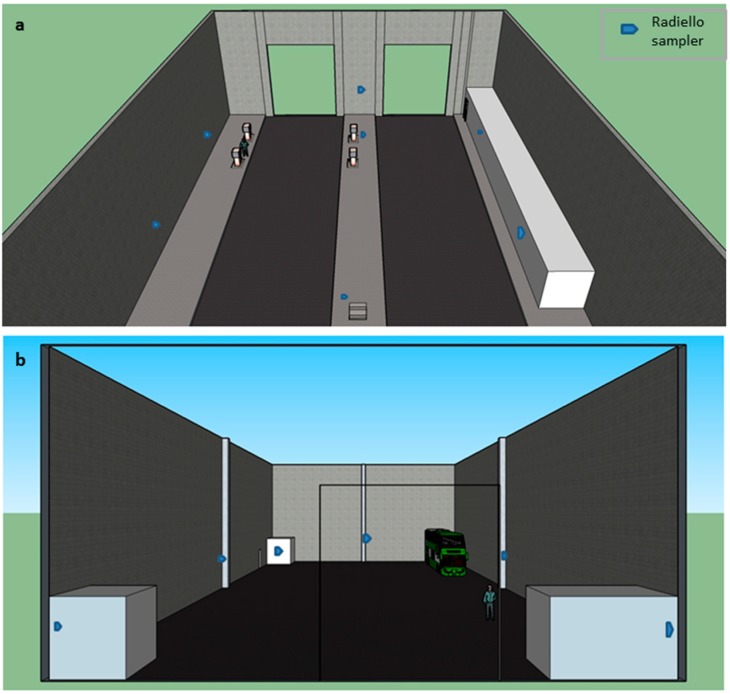
Radiello passive sampler positions in the (**a**) fuel bay and (**b**) workshop. Passive samplers were positioned equidistant as possible, at 2 m heights. (Overhead roofs and front walls have been made transparent for viewing purposes. Offices are displayed as white boxes). The fuel bay is adjacent to the workshop at the bus depot.

The buses are fueled with standard 500 ppm diesel, in the refueling bay on site. The refueling bay consists of four diesel pumps, with four full time employed personnel. The refueling bay is undercover, with large 3 m high doors on either end of the bay. Bus engines continue running while refueling, thus exhaust fumes are present in the bay as well as vapors from refueling. In close proximity to the refueling bay (Figure 1a) is a large enclosed workshop where maintenance and repairs take place (Figure 1b). There is very little ventilation in the workshop, and all filters and extraction fans on site are out of order in both workspaces.

### 2.2. Study Sample

The occupationally exposed group consisted of fuel bay attendants (FBA) from the refueling bay (*n* = 4) and diesel auto mechanics (AM) from the bus workshop (*n* = 16). Only full time employees were considered for this study. The demographic information provided by the bus company is illustrated in Table 2. All employees worked a standard 8 hours per day, 5 days a week.

The majority of the employees at the site were male (only one auto-mechanic at the workshop was female). Overall, the majority of the employees did not smoke (75%). As can be seen in Table 2 there is a very wide range of employee ages (24 to 63 years) and exposure duration (from 1 to 41 years).

**Table 2:** Demographic data of participants in the fuel bay attendants (FBA) and auto-mechanics (AM). Data supplied by bus operating company.

Participant ID	Workplace	Gender	Smoker	Age	Employment Duration
				(years)	(years)
FBA1	Fuel Bay	Male	No	27	5
FBA2	Fuel Bay	Male	No	45	10
FBA3	Fuel Bay	Male	No	59	33
FBA4	Fuel Bay	Male	Yes	56	37
AM1	Workshop	Male	Yes	27	1
AM2	Workshop	Male	Yes	35	1
AM3	Workshop	Male	No	25	2
AM4	Workshop	Male	No	26	2
AM5	Workshop	Male	No	24	2
AM6	Workshop	Male	No	25	3
AM7	Workshop	Male	No	29	4
AM8	Workshop	Male	No	36	5
AM9	Workshop *	Female	No	47	8
AM10	Workshop *	Male	Yes	40	10
AM11	Workshop	Male	No	41	10
AM12	Workshop	Male	Yes	51	11
AM13	Workshop	Male	No	40	16
AM14	Workshop	Male	No	38	16
AM15	Workshop	Male	No	49	28
AM16	Workshop *	Male	No	63	41

* Employee based within an enclosed office inside the workshop.

### 2.3. Passive Sampling Strategy

Sampling and monitoring occurred during the winter, as many studies using both active and passive sampling strategies have established that BTEX concentrations are elevated in winter as compared to other seasons [4,25,26,27]. Johannesburg, located on the interior plateau of the country, experiences cold, dry winters, with temperatures ranging from −3 to 19 °C. A Luft Weather Sensor, with built-in temperature, humidity, wind speed and wind direction sensors, was deployed within the refueling bay, as there are garage doors that can allow for in/out flow of fresh air.

Radiello passive air samplers were arranged in both the refueling bay and workshop (Figure 1), where emissions were considered to be at their maximum. These samplers are considered reliable in both indoor and outdoor environments, and following the European standard (EN 13528-2), the Radiello passive samplers were used to analyze the risk exposure of BTEX in this situation [28]. Concentrations as low as 2 μg·m^−3^ may be measured with the samplers, with an error not exceeding 0.1 μg·m^−3^. The samplers were deployed for 14 days as prescribed by the manufacturer. The BTEX passive samplers consist of an absorbing cartridge, which is placed in a micro-porous polyethylene membrane surface (50 mm long micro-porous cylinder; 16 mm external diameter; 300 mg of 40–60 mesh Carborograph 4) [28,29]. Each cartridge was secured to a triangular polycarbonate supporting plate. The sampling plates and cartridges were not placed within protective chambers, as wind speeds were low enough to avoid error [30] (average wind speeds were 2 m·s^−1^ in the refueling bay and workshop, as they are undercover sites). As outlined by Gallego *et al.* [25], each sample was labelled, and initial and final sampling times were recorded. As soon as the sampling process was over, tubes were returned to their protective containers and sent to a laboratory for analysis (ChemTech Labs, Johannesburg, South Africa). Tubes containing the samples were stored in a dark, cool box. As advised by the laboratory, leaded pencils were avoided as to preclude any contamination of the samples.

### 2.4. Risk Characterization

As has been shown in many studies, inhalation risk analysis is vital in order to determine the potential exposure of employees [15,19,21,31]. Both cancer risk and hazard risk calculations (associated with the inhalation of air pollutants) were done for employees to evaluate the potential effect of BTEX on human health. Individual calculated cancer risk and hazard risk values were compared with the United States Environmental Protection Agency (US EPA) acceptable standards.

To calculate cancer risk (CR) Equation (1) was applied, while Equation (2) was used to evaluate the non-carcinogenic hazard quotient (HQ):

*Cancer Risk (CR) = Lifetime Average Daily Dose (LADD) × Slope Factor* (1)

*Hazard Quotient (HQ) = Lifetime Average Daily Dose (LADD)/reference dose* (2)

Inhalation slope factor (SF) [benzene 0,0273 (mg/kg/day)^−1^] and reference dose (RfD) standard values were used [benzene 0,00855 mg∙kg^−1^∙day^−1^, toluene 1.43 mg∙kg^−1^∙day^−1^ and xylenes 0,029 mg∙kg^−1^∙day^−1^] [22,31].

To calculate the Lifetime Average Daily Dose (LADD) of employees, Equation (3) was utilised:

*LADD = (C.CF.IR.EF.ED)/(BW.AT)* (3)

where C is the contaminant concentration (average concentrations used from passive samplers) (μg/m^3^); CF is the conversion factor (1 mg/1000 μg); IR is the inhalation rate (US EPA standard) (20 m^3^/day); EF is the exposure frequency (days/year); ED is the exposure duration (years); BW is body weight (US EPA standard) (70 kg); and AT is the averaging time (exposure averaged over life time/average life expectancy for male and female) (days).

Demographic data were provided by the operations manager, in order to provide information pertaining to individual input variables such as age, sex, exposure frequency and exposure duration (Table 2). Where data were limited, US EPA standard values were used for body weight (70 kg for males and 60 kg for females) and inhalation rate (20 m^3^/day) [31]. In order to calculate the exposure frequency, standard values were used (all employees worked a standard five days, eight hours per day and received a minimum of 21 days leave per annum). More detailed evaluation of confounding factors, such as smoking habits and home conditions, was beyond the scope of this study.

## 3. Results

### 3.1. BTEX Monitoring

BTEX concentrations from passive samplers are shown in Table 3. Averages of these concentrations have been used to analyse the potential risk of employees (*i.e.*, contaminant concentration (Equation (3)). The average benzene ambient concentration results in the general fuel bay and workshop areas as well as the workshop offices (Table 3) pose a potential cancer risk for employees, as the World Health Organisation (WHO) states that benzene is a known human carcinogen and thus no safe level of exposure can be recommended. Regarding the workshop, there is a statistically insignificant difference between concentrations found within individual offices and the general area of the workshop; however, concentrations are slightly lower in the offices. One important factor to note is the higher levels of toluene and xylenes in the general area of the workshop (maximum concentrations of 11.93 and 13.12 ppb, respectively), as compared to all other workspaces at the study site.

**Table 3:** Average BTEX concentrations (in ppb) from Radiello passive samplers in the refueling bay and workshop (average atmospheric temperature during the monitoring period was 14.3 °C; six samplers were placed in each workspace).

	Benzene	Toluene	Ethylbenzene	Xylenes
**Fuel Bay General Area**				
Geometric Mean	1.21	2.26	0.57	3.52
Max	1.26	2.43	0.87	4.97
Min	1.16	2.13	0.42	2.09
s.d	0.15	0.57	1.06	2.72
**Workshop—General Area**				
Geometric Mean	1.41	3.22	0.64	3.97
Max	1.65	11.93	3.35	13.12
Min	1.25	2.33	0.41	2.25
s.d	0.66	9.82	7.30	10.03
**Workshop—Offices**				
Geometric Mean	1.38	2.76	0.67	4.10
Max	1.48	3.00	0.96	4.79
Min	1.29	2.46	0.50	2.84
s.d	0.32	1.08	1.05	4.40

### 3.2. Health Risk Assessment

According to the US EPA, a cancer risk above 1 × 10^−6^ is unfavourable, as it significantly increases carcinogenic potential in humans. All employees exceed the critical guideline value at this study site (Table 4). Of particular concern are participants FBA4 and AM16 (Table 4), as they have a potential risk of 1 in 1000 chance of developing cancer (1 × 10^−3^). These two employees have been employed the longest, at 37 and 41 years, respectively.

**Table 4:** Lifetime potential cancer risk for individual participants from exposure to benzene. The potential risk of 1 × 10^−5^ = 1 in 100,000; 1 × 10^−4^ = 1 in 10,000; and 1 × 10^−3^ = 1 in 1000 is based on the probability of developing cancer in a population sample.

Participant ID	Cancer Risk
FBA1	1.37 × 10^−4^
FBA2	2.74 × 10^−4^
FBA3	9.03 × 10^−4^
FBA4	1.01 × 10^−3^
AM1	3.24 × 10^−5^
AM2	3.24 × 10^−5^
AM3	6.47 × 10^−5^
AM4	6.47 × 10^−5^
AM5	6.47 × 10^−5^
AM6	9.71 × 10^−5^
AM7	1.29 × 10^−4^
AM8	1.62 × 10^−4^
AM9	2.72 × 10^−4^
AM10	3.07 × 10^−4^
AM11	3.24 × 10^−4^
AM12	3.56 × 10^−4^
AM13	5.18 × 10^−4^
AM14	5.18 × 10^−4^
AM15	9.06 × 10^−4^
AM16	1.39 × 10^−3^

A hazard quotient (HQ) is a measure of potential overall hazard risk. A HQ of ≥1 is considered as an “adverse non-carcinogenic effect of concern”; while a value of < 1 considered an “acceptable level” [19]. Thus, xylene concentrations at the site pose a low potential hazard risk, and are within acceptable standards (Table 5). However, benzene and toluene HQ are high, implying potential adverse health effects to employees.

**Table 5:** The hazard quotient (HQ) for benzene, toluene and xylenes; indicating the potential hazard risk to employees on exposure to compounds. An HQ >1 is considered an adverse non-carcinogenic effect of concern. HQ levels ≥1 are in bold for individual participants.

Participant ID	Hazard Quotient		
	Benzene	Toluene	Xylenes
FBA1	0.717	**1.588**	0.046
FBA2	**1.433**	**3.176**	0.092
FBA3	**4.731**	**10.480**	0.305
FBA4	**5.304**	**11.751**	0.342
AM1	0.170	0.452	0.012
AM2	0.170	0.452	0.012
AM3	0.339	0.904	0.024
AM4	0.339	0.904	0.024
AM5	0.339	0.904	0.024
AM6	0.509	**1.356**	0.036
AM7	0.678	**1.808**	0.048
AM8	0.848	**2.260**	0.060
AM9	**1.424**	**3.559**	0.115
AM10	**1.610**	**3.848**	0.124
AM11	**1.695**	**4.521**	0.120
AM12	**1.865**	**4.973**	0.132
AM13	**2.712**	**7.233**	0.192
AM14	**2.712**	**7.233**	0.192
AM15	**4.746**	**12.659**	0.335
AM16	**7.261**	**15.778**	0.509

**Figure 2:**
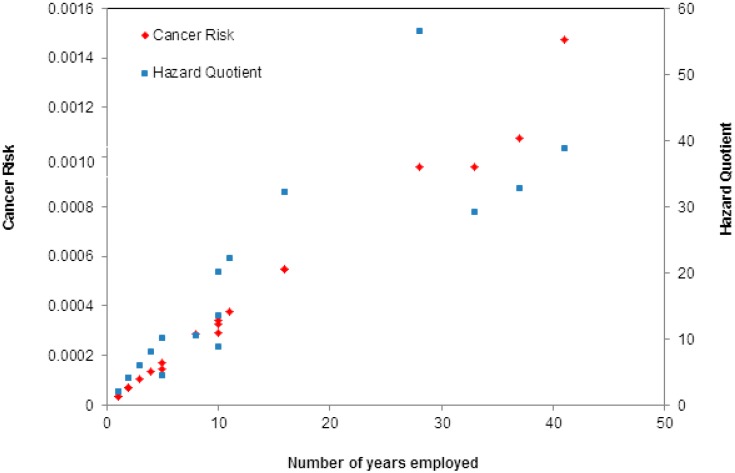
Cancer risk and hazard quotient (combined benzene, toluene and xylene hazard quotients) as compared to number of years employed.

Employees that have worked for more than 30 years are especially at risk to adverse non-carcinogenic effects (*i.e.*, FBA3-4 and AM15-16). This is further illustrated in Figure 2, where it is evident that with increasing work duration, there is a significant increase in both potential cancer and hazard risks. In addition to exposure duration playing a role in potential risk, placement within the workshop also plays a role. Thus, the range of risk is large at times, where inhalation exposures differ.

## 4. Discussion

### 4.1. BTEX Monitoring

The average benzene concentrations from the passive samplers measured at this site (Table 3) are significantly higher than the average concentrations of benzene measured in the atmospheric air of many European cities (e.g., Belgium, Greece, Italy, *etc.*). However, concentrations from the European studies are annual average concentrations, where concentrations may be decreased in summer months. Higher concentrations of benzene are generally reported in winter months, as a lower average ambient temperature has been noted to contribute to the accumulation of pollutants in the atmosphere as there is reduced movement of air masses in the upper atmosphere [29]. This study was conducted during the winter season, and consequently, results may be elevated relative to annual means. Furthermore, the ambient measurements from the European cities may not be site specific, compared to refuelling bays and workshops where levels may be intensified, as they may be generalised ambient air quality monitoring campaigns. However, mean benzene concentration in the fuel bay and workshop general areas, as well as the workshop offices (1.20; 1.40; 1.37 ppb, respectively) exceeds the US EPA’s inhalation exposure reference concentration (1.2 ppb) and the chronic inhalation reference concentration (3 × 10^−5^ ppb), as well as the WHO air quality guideline (0 ppb).

When buses are re-fuelled, diesel vaporisation and diesel exhaust emissions from the idling buses contribute to the increased benzene concentrations. The fuel bay serves as a source of diesel fuel in this study, however average benzene emissions are higher in the workshop than the fuel bay. There is not sufficient evidence to explain this relationship, but it does reveal the importance of diesel exhaust emissions when considering human health in occupational environments, as ventilation and extraction mechanisms are not in place at the study site. It should be noted that the workshop is generally more active with idling buses being repaired by auto-mechanics throughout the day, with spray painting activities also occurring, which may increase BTEX concentrations.

Although adjustments have been made to reduce the percentage volume of benzene in diesel fuel there is still a global trend of increased benzene emissions near fuel stations [19,24,29,32]. Edokpolo *et al.* [22] postulate that vaporisation inside fuel stations is the main sources of benzene in the atmosphere nearby. In a study conducted by Karakitsios *et al.* in Greece, similar studies indicated that even in developed countries where vapour recovery systems exist, filling station attendants are still exposed to high benzene concentrations (5–16 ppb) [13]. Benzene levels were also found to be directly proportional to volume of fuel dispensed [12,13].

### 4.2. Quantitative Risk Analysis

Results from the lifetime cancer risk estimation on employees at the bus depot show that on average there is a 3.78 × 10^−4^ cancer risk, corresponding to an average chronic daily intake of 1.38 × 10^−3^ mg·kg^−1^·day^−1^ of benzene exposure. This implies that, on average, there is 3-in-10,000 chance that employees at the site may develop cancer in their lifetime, with some employees experiencing even higher probabilities (Table 4). The lifetime cancer risk thus exceeds the US EPA standard of 1 × 10^−6^ for all employees (Table 4). Health risk assessments conducted in a wide variety of environments reiterate this finding. Studies indicate that cancer risks of sample groups exposed to benzene concentrations generally exceed the US EPA cancer risk limit [32,33,34,35,36,37].

It was determined by Guo *et al.* [38] that inhalation exposure to benzene accounts for more than 40% of cancer risks for various indoor environments. This finding was confirmed in other studies where benzene baseline blood levels were higher in groups exposed to constant BTEX emissions, compared to those that are not exposed. It was found that benzene levels in blood were directly proportional to benzene concentrations in the atmosphere, specifically in fuel stations [9,33]. Romieu *et al.* [9] also determined that the blood baseline benzene levels of fuel attendants did not increase over the work shift as expected. This was attributed to chronic level of exposure, and not short term exposure, thus determining potential lifetime cancer risks is essential in high risk areas.

At this study site, the majority of the employees did not smoke, however many studies have confirmed that long term exposure to volatile organic compounds from diesel exhaust emissions increased the cancer risk among smokers and non-smokers alike [39]. Weisel [40] found that benzene inhalation exposure in occupational settings may be increased in employees who smoke. However, Oesch *et al.* [41] suggested that smokers are sporadically less affected to BTEX inhalation as smoking has a detoxifying effect. Regrettably, this study could not take into account the exact effect of smoking when exposed to high levels of BTEX, and analyze the potential cancer and hazard risks. However, whether employees smoked or not, it was determined that long term exposure to BTEX increased hazard in high-risk areas, such as fuel bays, repair centers and spray painting centers [41]. This was also noted in the current study, where higher concentrations of BTEX were noted in the workshop (where auto-mechanics operate) as compared to the fuel bay (Table 3). This is further illustrated in Table 5 where a greater proportion of auto-mechanics face potential hazard risks, as compared to fuel bay attendants. Colman Lerner *et al.* [32] showed that when compared to many different occupational settings, auto-mechanics and car painting centers showed the highest levels of VOCs, including BTEX.

In both the fuel bay and workshop, there is very little ventilation and no filtration/extraction fans. However, the fuel bay experiences some natural ventilation as air flow occurs through the open doors. This is not the case in the workshop where little to no natural ventilation occurs. This further increases CR and HQ estimates. In Montréal, Canada, BTEX exposure among auto mechanics and painters were within standards; levels were low and did not cause a hazard or cancer risk. However, when both mechanical and natural ventilation systems were used, BTEX concentrations were significantly reduced, as opposed to only natural ventilation system usage [18]. This indicates the urgent need for mechanical ventilation systems to be fixed at the study site and to be maintained properly for such working environments in general.

In addition to lack of ventilation and extraction systems, exposure duration also plays a major role in potential lifetime risks, to both hazard and cancer. Results in this study illustrate that with continuous exposure, CR and HQ exponentially increase, especially for personnel employed for over 30 years (Figure 2). Das *et al.* [8] argued that long term exposure led to increased hazard risk. The researchers found that health related signs were commonly observed in workers employed for more than 5 years at retail petrol pump stations. Workers suffered from neurological symptoms (such as headaches) and eye irritations at these sites. In another study, mean lifetime cancer risks for workers exposed to benzene and ethyl-benzene for 30 years in gas stations in Bangkok, Thailand, was estimated to be 1.75 × 10^−4^ and 9.55 × 10^−7^, respectively [19]. Exposure to these VOCs significantly led to fatigue. These findings are similar to results found in this study (Table 4), where exposure to benzene yielded a mean lifetime cancer risk of 2.19 × 10^−4^.

In addition to smoking habits, ventilation systems and exposure duration, proximity to high levels of BTEX also affects potential risk estimations. Thus, findings revealed that employees placed within offices, further away from direct exhaust emissions, were exposed to slightly lower concentrations of BTEX, and thus experienced lower hazard quotients (Table 5). McKenzie *et al.* [42] determined that the distance from gas wells was significantly associated to the health risks associated with VOC exposure. It was shown that residents < 1km from the gas well were at higher risk of chronic and acute health risks [42]. This was also found by Karakitsios *et al.* [12] in Epirus, Greece, where cancer risk for the general population in close proximity to filling stations increased by 3% to 21%. Thus, many different factors contribute to increased inhalation exposure, and inevitably lead to increased potential health risk.

## 5. Conclusions

The health risk assessment conducted at this site indicates that employees are at risk to carcinogenic effects, and the CR for all employees exceeds the US EPA cancer limits. BTEX concentrations are higher than in other comparative studies. Lack of both mechanical and natural ventilation systems, especially in the workshop, exacerbates the exposure of auto-mechanics and fuel bay attendants. However, despite these findings, confounding factors; such as smoking history, personal medication usage and baseline health status; were not accounted for in this study, and this may skew risk estimations. Future research design should avoid, and/or take into account these confounding factors, as they may affect CR and HR risk estimations.

Overall however, results indicate that ambient concentrations and health risk estimates are generally above international guidelines at the site, and are a matter of concern. This study demonstrates that health risk assessments in conjunction with medical studies (e.g., Keretetse *et al.* [33]) are highly necessary in South Africa and elsewhere, especially in the developing world, to serve as a foundation to amend national exposure limits which will protect employees in high risk jobs.
